# Effect of the Carbon Support and Conditions on the Carbothermal Synthesis of Cu-Molybdenum Carbide and Its Application on CO_2_ Hydrogenation to Methanol

**DOI:** 10.3390/nano12071048

**Published:** 2022-03-23

**Authors:** Ana Belén Dongil, Elodie Blanco, Juan José Villora-Picó, Antonio Sepúlveda-Escribano, Inmaculada Rodríguez-Ramos

**Affiliations:** 1 Instituto de Catálisis y Petroleoquímica, CSIC, C/Marie Curie 2, Cantoblanco, 28049 Madrid, Spain; irodriguez@icp.csic.es; 2 Departamento de Ingeniería y Gestión de la Construcción, Pontificia Universidad Católica de Chile, Santiago 2530388, Chile; 3 Departamento de Ingeniería Química y Bioprocesos, Pontificia Universidad Católica de Chile, Santiago 2530388, Chile; 4 Millenium Nuclei on Catalytic Processes towards Sustainable Chemistry (CSC), Santiago 2530388, Chile; 5 Laboratorio de Materiales Avanzados, Departamento de Química Inorgánica—Instituto Universitario de Materiales de Alicante, Universidad de Alicante, Apartado 99, 03080 Alicante, Spain; jj.villora@ua.es (J.J.V.-P.); asepul@ua.es (A.S.-E.)

**Keywords:** CO_2_ hydrogenation, molybdenum carbide, copper, methanol, carbon

## Abstract

The synthesis of methanol by carbon dioxide hydrogenation has been studied using copper-molybdenum carbides supported on high surface area graphite, reduced graphene oxide and carbon nanotubes. The synthesis conditions and the effect of the support were studied. The catalysts were prepared in situ using H_2_ or He at 600 °C or 700 °C. Both molybdenum carbide and oxycarbide were obtained. A support with less reactive carbon resulted in lower proportion of carbide obtained. The best results were achieved over a 5 wt.% Cu and 10 wt.% Mo on high surface area graphite that reached 96.3% selectivity to methanol.

## 1. Introduction

Methanol is a key compound of chemical industry with a current production of 98 MTm/year which is expected to increase up to 500 MTm/year by 2050. It has applications in the production of polymers or fuels among others, and its use in fuel cells is promising since it is a stable molecule easy to transport and with high energy density [1,2]. However, methanol is currently produced mainly from fossil sources, representing around 10% of CO_2_ emissions from industry. Green methanol production can be achieved through the use of biomass as source or CO_2_ captured from renewable sources, i.e., bioenergy, and renewable hydrogen.

Moreover, there are large emission sources within the steel, power, or concrete industry which can benefit from CO_2_ capture and thermocatalytic conversion, especially as CO_2_ emissions taxes increase, this making the carbon capture and use more interesting [3].

Hence, the interest on efficient catalytic processes for CO_2_ to methanol conversion is evident. The first tested catalysts were those already used in the hydrogenation of syngas of the type Cu/ZnO/Al_2_O_3_, for which typical industrial conditions are 230–280 °C and 50–120 bar. Copper as active phase commonly leads to deactivation due to its high tendency to sintering and low hydrothermal stability. More recently, molybdenum carbide has been successfully employed as an inexpensive and active alternative with ability to activate both CO_2_ and H_2_. Moreover, combined with a second metal such as copper, it can reach high selectivity to methanol, which has been compared to noble metals such as palladium [4,5].

The synthesis of molybdenum carbide over several supports has been previously reported using hydrogen/hydrocarbon at 600–800 °C resulting in different extent of carburization [6,7,8]. Unsupported molybdenum carbides are generally covered by carbon, and the obtained surface areas of around 30 m^2^/g [9] are not optimal for catalysis. Alternatively, some organic precursors have been employed; however, the active phase generally remains covered with carbon from the precursor [10,11,12].

As a cleaner alternative, carbothermally generated carbide on carbon supports such as activated carbon, graphite, or carbon nanofibers has been reported by us and other groups [13,14,15,16]. The synthesis is influenced by the experimental conditions and the nature of the support.

Hence, we have studied the synthesis of copper-molybdenum carbide catalysts over different carbon supports (a commercial carbon support of high surface area, reduced graphene oxide, and carbon nanotubes), aiming at using milder synthesis conditions. The effect of different synthesis atmosphere (H_2_ or He) and temperature (600 or 700 °C) on the physico-chemical and catalytic performance has been evaluated.

## 2. Materials and Methods

### 2.1. Catalysts Synthesis

Supported metal carbides catalysts were prepared by wetness impregnation of the precursors followed by a carburization treatment. The supports employed were a commercial high surface area graphite (HSAG400 from Timcal Graphite, Timcal, SA, Switzerland), a lab-prepared reduced graphene oxide, and commercial carbon nanotubes Baytubes C150 HP (Bayer Materials Science, Pittsburgh, PA, USA) that will be labelled as H (S_BET_ = 399 m^2^/g), rGO (S_BET_ = 505 m^2^/g) and CNT (S_BET_ = 264 m^2^/g), respectively. The synthesis of the rGO support has been thoroughly described before [17]. In brief, graphenic materials were prepared by the treatment of graphite oxide (GO) at high temperature. The starting graphite used to prepare GO was natural graphite powders of grain size 10 mesh (supplied by Alfa Aesar, Thermo Fisher Scientific, Lancashire, UK, purity 99.8%). The conditions used for the reduction and exfoliation of the graphite oxide can be summarised as follows. (A) a N_2_ flow (87 mL min^−1^) was used. The heat treatment was 10 °C min^−1^ up to 250 °C. Then, this temperature was kept for 30 min and then increased from 250 to 700 °C at 10 °C min^−1^, where it was maintained for 30 min. Metal carbides were prepared by wet co-impregnation on the carbon supports with the corresponding metal precursors. Aqueous solutions of the Mo precursor, (NH_4_)_6_Mo_7_O_24_ (99% from Aldrich, MO, USA) and Cu precursor Cu(NO_3_)_2_^.^3H_2_O (99% from Aldrich), were employed with the calculated weight to obtain the target metal loading, and after 6 h maturation, samples were dried overnight at 100 °C. The carburization was carried out for 2 h under pure H_2_ or He, at 500, 550, 600, or 700 °C, (5 °C min^−1^). The Mo and Cu loadings were 10 wt.% and 5 wt.% in all cases. In this way, eight samples were prepared and labelled according to the support (H, rGO, and CNT) and carburization conditions (when H_2_ and/or 600 °C were used the reactions were omitted): CuMoxC/H500; CuMoxC/H550; CuMoxC/H; CuMoxC/H700; CuMoxC/H-He; CuMoxC/H700-He, CuMoxC/rGO, and CuMoxC/CNT.

### 2.2. Characterization

N_2_ adsorption isotherms were obtained at −196 °C using a 3Flex instrument from Micromeritics to assess the textural properties of the samples. About 100 mg of sample previously degassed at 120 °C for 4 h under vacuum. The surface area was calculated from the adsorption branch in the range 0.02 ≤ p/p0 ≤ 0.25, using the Brunauer–Emmett–Teller (BET) model.

In situ X-ray diffraction (XRD) patterns were obtained in a reaction chamber (Anton Par XRK900, Graz, Austria) by flowing H_2_ or N_2_. The 2θ range was between 4° and 90°, with a step of 0.04 °/s, using a Polycristal X’Pert Pro PANalytical diffractometer with Ni-filtered Cu Kα radiation (λ = 1.54 Å) operating at 45 kV and 40 mA.

Photoelectron spectra (XPS) were recorded using an Escalab 200 R spectrometer equipped with a hemispherical analyser and using non-monochromatic Mg K X-ray radiation (hν = 1253.6 eV). The samples were carburized as described in Section 2.1 and poured into an octane solution to avoid oxidation before transferring to the outgassing chamber. Prior to the experiments, samples were outgassed in situ for 24 h to achieve a dynamic vacuum below 10–10 mbar. The binding energy (BE) was measured by reference to the C 1s peak at 284.6 eV, with an equipment error of less than 0.01 eV in the energy determinations. The surface atomic ratios were estimated from the integrated intensities of Mo 3d, Cu 2p, C 1s, and O 1s lines after background subtraction and correction by the atomic sensitivity factors. The spectra were fitted to a combination of Gaussian–Lorentzian lines of variable proportions.

The thermogravimetric analyses (TGA) were performed with an TA Instruments SDT Q600 TA system from 30 °C to 700 °C, at 5 °C min^−1^, in H_2_ or He flow (100 cm^3^ min^−1^, STP). The evolved gases from the TGA were monitored by mass spectrometry using a ThermoStar GSD 301 T3 instrument (filament 150 °C, SEM and emission detector at 950 mV). The masses analysed were 2, 14, 15, 16, 17, 18, 28, and 44.

Raman spectra were obtained with a spectrometer Jobin Yvon LabRAM HR equipped with a microscope. In order not to heat the graphene monolayer, a power of 2 mW and a source of excitation at 514 nm from an argon laser were used. The resolution of the equipment is 1 cm^−1^, and the laser beam was focused onto the sample with a 100× objective. The laser intensity at the sample was kept below the threshold for any laser-induced changes in the Raman spectra and electrical transport characteristics.

Temperature-programmed desorption of carbon dioxide (TPD-CO_2_) was carried out with a combination of a thermal conductivity detector (TCD) and a mass spectrometer (Cirrus 2, MKS Spectra product, Cheshire, UK) using a 3Flex device (micromeritics). Typically, around 50–75 mg of the sample was first carburized in situ under H_2_ flow (50 mL min^−1^) at 600 °C (5 °C min^−1^) for 2 h. The reactor was then cooled at 100 °C and purged for 1 h under He (50 mL min^−1^) prior adsorption of CO_2_ (50 mL min^−1^) for 30 min. The sample was finally cooled to 45 °C and purged for 2 h under He (50 mL min^−1^). Finally, desorption was carried out by heating under the same He flow at 5 °C min^−1^ from 45 to 600 °C while monitoring the following *m*/*z* fragments: 2, 15, 18, 28, and 44 related to H_2_, CH_4_, H_2_O, CO, and CO_2_ respectively.

### 2.3. Reaction

The evaluation of the gas phase catalytic performance was carried out in a stainless steel fixed-bed flow reactor of 3/8″ internal diameter. The catalysts (0.5 g) were placed in the reactor and in situ carburized in pure H_2_ or He, by increasing the temperature up to 100 °C, keeping this temperature for 1 h and then raising it up to 500, 550, 600, or 700 °C, with a heating rate of 5 °C min^−1^. The treatment at this temperature was carried out for 2 h. Finally, catalysts were cooled under H_2_ to the reaction temperature, 150 °C. The reactor was pressurised at 20 bar using a reactant mixture composed of (CO_2_:H_2_:He = 10:30:60 vol), and the total flow during reaction was 30 mL min^−1^. The reactants and products were analysed by gas chromatography (Varian CP 3400, Varian, CA, USA) with FID and TCD detectors, fitted with SupelQ Plot and 60/80 Carboxen-1000 columns, respectively. The reaction conditions allowed to maintain conversions in all the experiments to assume differential reactor. The experimental error was calculated by repeating some of the experiments and resulted in the range of ±1%. The carbon balance was over 95% in all cases, and blank tests resulted in conversion below 0.5%. The conversion and products selectivity were obtained according to the following equations:

(1) $$X_{{\mathrm{CO}}_{2}}=\frac{\sum_{i}ni\times \mathrm{moli}}{\sum_{i}ni\times \mathrm{moli}+{\mathrm{molCO}}_{2}-\mathrm{un}}\times 100$$

(2) $$S_{i}=\frac{ni\times \mathrm{moli}}{\sum_{i}ni\;\times \;\mathrm{moli}}\times 100$$

where:

n_i_: number of carbon atoms of product i.
mol_i_: number of moles of product i.
mol CO_2_-un: mol of unreacted CO_2_.

## 3. Results

### 3.1. Effect of the Support and Synthesis Conditions on the Molybdenum Crystal Phases

**Figure 1:**
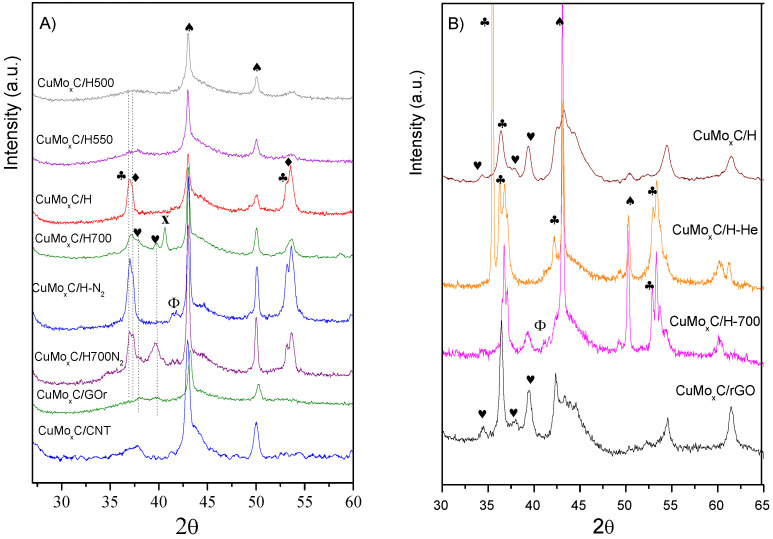
(**A**) In situ XRD patterns under H_2_ or inert atmosphere (N_2_) of the catalysts up to 600 °C or 700 °C. (**B**) XRD patterns of spent catalysts. Symbols: ♥ Mo_x_C ♣ MoO_x_C_y_, ♦ MoO_2_, x Mo, ♠ Cu, Φ CuO.

The molybdenum crystal phases obtained have been identified by in situ XRD under H_2_ or N_2_, and the patterns are shown in Figure 1A. It can be observed that the support and the reaction conditions clearly influence the XRD pattern.

While diffractions ascribed to molybdenum phases are barely insinuated at around 37.1° for CuMo_x_C/H-500 and CuMo_x_C/H-550, the catalyst CuMo_x_C/H showed diffractions with maxima at 2θ of 36.9° that seem to include another contribution at 37.1° and at 53.1°. According to our previous results combining XANES, XPS, and XRD characterization, the first peak may contain contributions of either MoO_x_C_y_ and/or MoO_2_. The possibility of molybdenum hydride species might also be considered, since they have been also identified and would appear at similar angles compared to MoO_x_C_y_ [4,6,18,19,20].

Further heating of this sample at 700 °C led to a pattern where diffractions at 2θ of 36.9°, 37.7°, and 39.5° are observed. In this case, it appears that the sample contains contributions due to MoO_x_C_y_, the first peak, and to those of the (002) and (101) planes of β-Mo_2_C hcp phase, the last two peaks. Moreover, diffractions at 2θ of 40.6° and 58.7° assigned to Mo^0^, which correspond to the (110) and (200) planes of the cubic structure of Mo^0^ (JCPDS 42–1120), respectively, are also observed.

When changing the synthesis conditions for CuMo_x_C/H to an inert atmosphere, the patterns are similar for both samples treated at 600 °C and 700 °C. They displayed peaks at 2θ of 36.7° and 37.1°, which again can be ascribed to MoOxCy, but the first diffraction can also include the contribution of the (111) plane of Cu_2_O [21]. Furthermore, the sample treated at 700 °C under inert atmosphere displayed an additional wide peak at 2θ of 39.4°, which can be attributed to MoO_3_. However, the detection of carbide and oxycarbide by XPS (see Table 1) could indicate that this peak also includes the diffraction of the β-Mo_2_C phase.

As long as the effect of the support is concerned, the sample prepared over reduced graphene oxide, CuMo_x_C/rGO, and treated under H_2_ at 600 °C, displayed a small hump with two insinuated peaks at 2θ of 37.7° and 39.6° which seem to indicate the presence of β-Mo_2_C and MoO_x_C_y_ phases. When the support for the metallic precursors was CNT, the resulting XRD showed a small contribution at around 2θ of 37.1° which may correspond to highly dispersed Mo phases, MoO_x_C_y_ and β-Mo_2_C.

Moreover, all the samples treated under hydrogen display diffractions at around 43.1° and 50.0°, which are assigned to the (111) and (200) planes of metallic copper (JCPDS 04-0836). Sample CuMo_x_C/H treated under inert atmosphere also showed diffractions at 2θ of 41.1° and 41.5° which correspond to Cu_2_O [21]. In addition, the absence of diffractions corresponding to copper carbide that would appear around 2θ of 36.0° indicates that under the employed conditions its formation is not favoured, as it was also verified by the XPS analyses [22,23].

**Figure 2:**
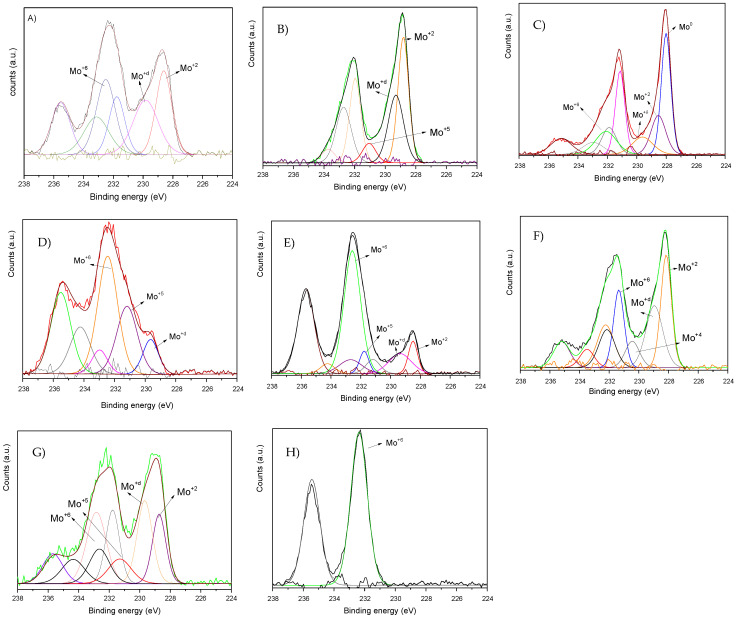
XPS Mo 3d Region of (**A**) CuMo_x_C/H550; (**B**) CuMo_x_C/H; (**C**) CuMo_x_C/H at 700 °C under H_2_; (**D**) CuMo_x_C/rGO; (**E**) CuMo_x_C/CNT; (**F**) CuMo_x_C/H under He at 600 °C; (**G**) CuMo_x_C/H under He at 700 °C; (**H**) Spent CuMo_x_C/H.

The oxidation states of Mo and Cu species and metal/C ratios were assessed by XPS of the carburized samples, and the Mo 3d_3/5_ region of each catalyst is shown in Figure 2. The Mo 3d region displayed several contributions which can be assigned to the following oxidation states: Mo^0^ (227.9 eV), Mo^+2^ (228.2–228.6 eV), Mo^+δ^ (228.9–229.8 eV), Mo^+5^ (231.0–231.2 eV), and Mo^+6^ (232.1–232.6 eV). The attribution of each binding energy is included in Table 1. Mo^+2^ corresponds to Mo with Mo-C bonds, while Mo^+5^ and Mo^+6^ are species of Mo_2_O_5_ and MoO_3_, respectively, due to incomplete carburization [24]. In addition, the Mo^+δ^ is an intermediate oxidation state between +4 and +2 that can be ascribed to MoO_x_C_y_.

From the XPS analyses some additional information can be gathered. Firstly, all the catalysts treated under H_2_ at 600 °C presented both Mo_x_C and MoO_x_C_y_ phases, but in different proportion, and the catalyst with the highest total surface atomic concentration of Mo_x_C and MoO_x_C_y_, ca. 90%, is the one prepared over HSAG. This catalyst also presented the highest surface atomic concentration of Mo_x_C. In contrast, when carbon nanotubes or reduced graphene oxide were used as supports, the contribution of MoO_3_ was more significant, suggesting a lower capacity to carburize the metal oxide.

On the other hand, among the catalysts prepared over HSAG at different temperatures under a H_2_ atmosphere, the one treated at 600 °C displayed the highest Mo_x_C + MoO_x_C_y_ contribution, while that synthetized at 700 °C showed the lowest value since Mo(0) is now present.

As long as the Mo/C ratio is concerned, all the catalysts showed similar ratio, except those treated at 700 °C under H_2_ for which the ratio was higher, which could be due to an improvement of the molybdenum dispersion due to particles rupture during H_2_ diffusion [8].

**Figure 3:**
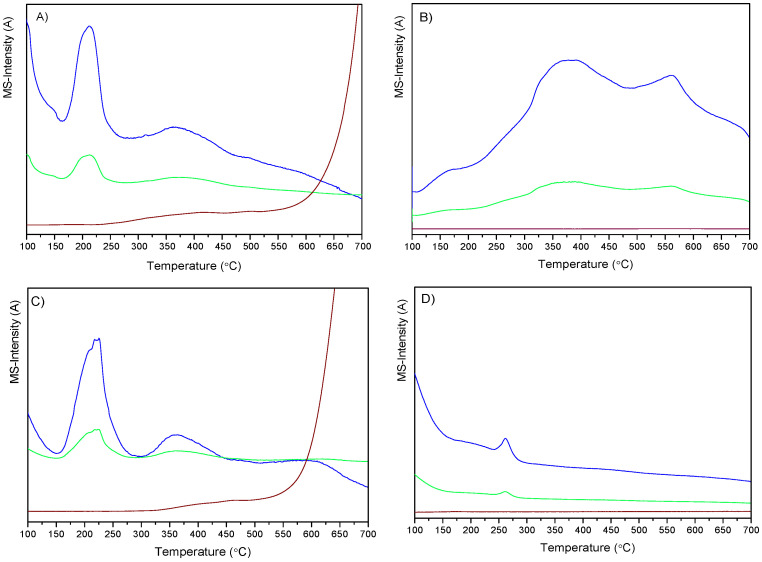
Mass profiles of H_2_-TPR of (**A**) CuMo_x_C/H, (**B**) CuMo_x_C/CNT, and (**C**) CuMo_x_C/rGO under H_2_ and (**D**) CuMo_x_C/H under He. *m*/*z* 18 (blue); *m*/*z* 17 (green) *m*/*z* 15 (wine).

The carburization process, under H_2_ or N_2_, was studied using a TG equipment coupled with a mass spectrometer to follow the evolved masses, *m*/*z*, and the results are shown in Figure 3.

The profiles of the experiments performed under H_2_ atmosphere for CuMo_x_C/H and CuMo_x_C/rGO are similar. For these samples, signals *m*/*z* 18 and 17 appear with maxima at 200 °C and 360 °C, for which the contribution of *m*/*z* 17 indicates that it is primarily due to H_2_O since the relative proportion of *m*/*z* 18 and 17 on the profiles matches with the ratios expected for water (*m*/*z* 17 is 20% of *m*/*z* 18). Moreover, the most intense peak is observed at the lowest temperature. The absence of a relevant contribution due to the evolution of NH_3_ and the fact that nitrogen was not detected by XPS suggest that evolved NH_3_ might have reduced NO_2_ resulting from the decomposition of the copper precursor, producing inert nitrogen.

The evolution of masses assigned to water at 200 °C is probably due to the reduction of copper oxides, while those at 370 °C would correspond to transformations of molybdenum oxides. *m*/*z* 15 was selected to assess CH_4_ evolution since the main fragment (*m*/*z* 16) could also include a minimal contribution from water or ammonia if any. This mass appeared as a broad and low intense hump in the range 300–500 °C, with maxima at 400 and 450 °C. At higher temperatures of around 600 °C for CuMo_x_C/H and 550 °C for CuMo_x_C/rGO, an intense *m*/*z* 15 contribution is observed. However, for the sample CuMo_x_C/CNT, the temperature at which the *m*/*z* 15 appears is higher, and this suggests that generating carbide carbothermally is more difficult over this support, in agreement with XPS.

When the same experiment was performed under inert atmosphere for the sample prepared over HSAG, the profile changed. For this sample, *m*/*z* 18 and 17 appeared with maximum at 260 °C, and *m*/*z* 16 was not detected.

Overall, the characterisation showed differences depending on the support and carburization conditions. The catalysts displayed MoO_x_C_y_/β-Mo_2_C and MoO_x_ phases, indicating that incomplete carburization took place under the studied conditions. Increasing the carburization temperature up to 700 °C did not result in a larger extent of carburization and, in contrast, Mo^0^ was obtained as the main phase.

Meanwhile, the favoured formation of carbide on HSAG compared to rGO might be due to the different structure of these supports. This feature was assessed by Raman spectroscopy included in Figure S1, which showed that the number of defects on the support such as edges, and estimated as the I_D_/I_G_ ratio, is higher on HSAG compared to rGO, 0.80 vs. 0.53. However, the same does not apply to CNT since for this support the high I_D_/I_G_ ratio, 1.81, is due to their rolling structure, while for H and rGO, the D-band mainly emerges from the presence of dangling bonds which can be more easily the source of carbon for carburization.

Finally, the samples prepared under inert atmosphere also presented the MoO_x_C_y_ phase but in a lower proportion than samples treated under H_2_. It was also observed that the contribution of molybdenum oxide was higher on the sample treated at 600 °C under inert atmosphere, and metallic copper was detected.

Regarding prior literature results, the carbothermal method using H_2_ or an inert atmosphere has been widely used for carbon supports. For example, for carbon nanofibers, the oxycarbide was already observed at 550 °C after synthesis under H_2_, which was then transformed into the β–Mo_2_C phase at 650 °C [25]. However, in other works using carbon nanotubes, the β–Mo_2_C phase was formed at 700 °C [26] or at 800 °C over activated carbon [16].

There are few reports in literature where formation of Mo(0) is described upon carburizing molybdenum on carbon supports using an H_2_ atmosphere [15,27]. We have recently reported the presence of a hydride phase on Cu-Mo_x_C catalysts and its plausible relation with the development of metallic Mo [6].

The formation of the different species may be simplified according to reactions (1) and (2) [19]

MoO_3_ + H_2_ → MoO_2_ + H_2_O (R1)

MoO_2_ + C* + H_2_ → MoO_x_C_Y_ + H_2_O → β–Mo_2_C + CO (R2)

Hence, the lack of enough available reactive carbon, C*, on the support and the presence of molybdenum hydride species can explain the reduction to Mo^0^, which is favoured under the H_2_ atmosphere.

### 3.2. Reaction Results

**Figure 4:**
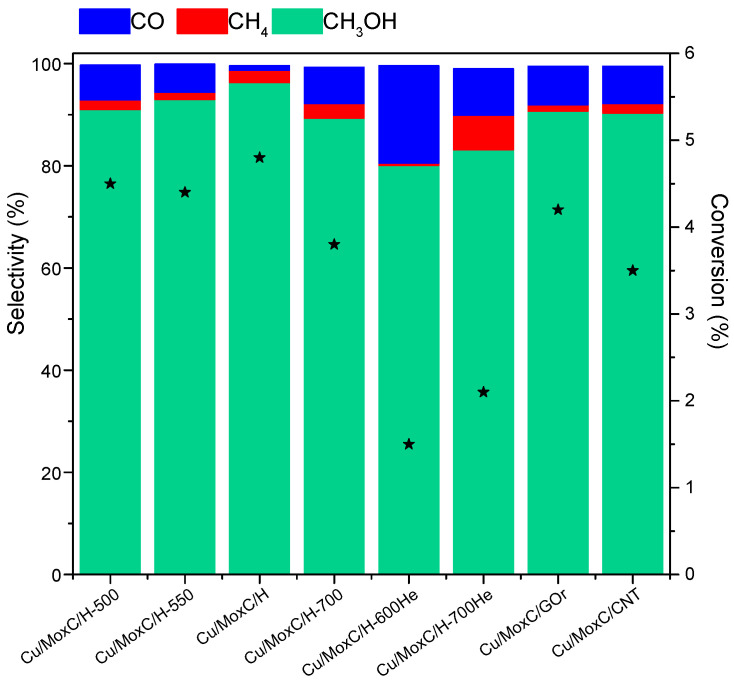
Conversion of CO_2_ (scatter in the right axes) and selectivity (in bars left axes) to CH_3_OH, CH_4_, and CO at 150 °C and 20 bar.

The conversion of CO_2_ was evaluated at 150 °C and 20 bar, and results are shown in Figure 4. The conversion achieved with the catalysts varied in the range 1.5–4.8%. The main product obtained with all the catalysts was methanol, and the selectivity to this product was in the range 80.1–96.3%, while methane and carbon monoxide were observed to some extent to depend on the catalyst.

If we compare the results obtained with CuMo_x_C/H carburized at different temperatures, 500–700 °C under hydrogen, changes in line with their different Mo species are noticed. The average conversion values indicate that the conversion obtained with CuMo_x_C/H is slightly higher than that obtained with the catalysts CuMo_x_C/H-500 and CuMo_x_C/H-550, in agreement with the higher proportion of Mo_x_C and MoO_x_C_y_ on CuMo_x_C/H.

The catalysts prepared over reduced graphene oxide and carbon nanotubes yielded lower conversion and selectivity than the catalysts prepared over HSAG under the same conditions. As expected, considering the lower extent of carburization, the catalysts prepared under inert atmosphere were less active and selective to methanol than the analogous samples prepared under hydrogen. However, the effect of the carburization temperature was different for the samples synthetized under inert or hydrogen atmosphere. Whereas for the catalysts prepared under hydrogen the catalytic performance was worse in terms of conversion and selectivity upon increasing the treatment temperature up to 700 °C, for the catalysts prepared under inert atmosphere the opposite trend was observed. The explanation can be found in the different phases obtained in the samples during the thermal treatment. As the characterization studies showed, the proportion of less active species on CO_2_ hydrogenation, i.e., Mo^0^ and MoO_x_, was different, which can explain straight forward the diverse catalytic behaviour.

Rodríguez et al. have studied the surface reactions occurring on Mo_2_C during CO_2_ hydrogenation, and they have found that the most abundant species were oxycarbides, which suggested a significant contribution on the catalytic performance [28]. It is probably that, under reaction, both phases, i.e., carbide and oxycarbide, coexist and are involved in a redox cycle where CO_2_ reacts with Mo_2_C through C-O interaction, producing oxycarbide and CO, which could eventually be reduced by hydrogen to obtain again Mo_2_C.

On the other hand, the conversion achieved with CuMo_x_C/H-700 is ca. 25% lower, which can be explained by the presence of Mo^0^ phase, which is not suitable for the activation of the C-O bond [29].

As regards to the effect of the support, the catalysts CuMo_x_C/rGO and CuMo_x_C/CNT are less active and less selective than CuMo_x_C/H. While the selectivity of both CuMo_x_C/rGO and CuMo_x_C/CNT is around 91% to methanol; the conversion reached 4.2 and 3.5%, respectively.

This can be explained by the higher proportion of molybdenum carbide phase on HSAG, as estimated by XPS.

**Figure 5:**
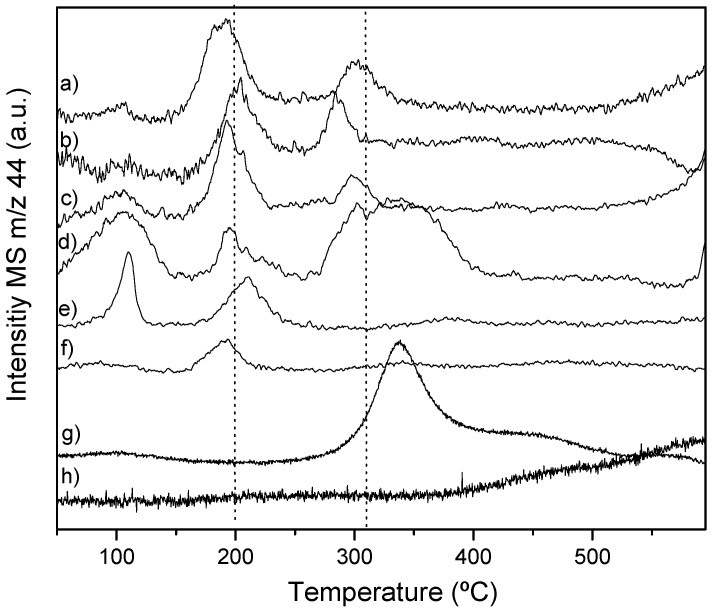
CO_2_-TPD profiles. (**a**) CuMo_x_C/H-700, (**b**) CuMo_x_C/H, (**c**) CuMo_x_C/H-550, (**d**) CuMo_x_C/H-500, (**e**) CuMo_x_C/rGO, (**f**) CuMo_x_C/CNT, (**g**) Cu/H and (**h**) Mo_x_C/H.

To further study the ability to activate CO_2_, CO_2_-TPD analyses were performed. The TPD profiles of the supports in Figure S2 confirmed that they do not adsorb CO_2_. Moreover, the evolution profile of *m*/*z* 28 for all the samples matches well the intensity of the secondary mass of CO_2_ (ca. 10%), and this suggests that for the studied systems, CO_2_ is only cleavage under H_2_ atmosphere during reaction conditions [30]. The evolution of *m*/*z* 44 ascribed to CO_2_ is shown in Figure 5. The contribution at temperatures around 100 °C can be assigned to physisorbed CO_2_. These adsorption sites can be due to Mo_x_C particles covered by carbon, C-Mo_x_C, and this would agree with the observed lower intensity of this peak upon increasing the carburization temperature. These sites would display a poor behaviour towards CO_2_ dissociation.

On the bimetallic catalysts prepared over HSAG, two desorption peaks at 193–210 °C and 285–303 °C are observed. For the sample carburized at 500 °C, the peak at the highest temperature comes along a wide shoulder which may be related to adsorption over oxidized species whose contribution, as observed by XPS, is higher.

The CO_2_-TPD profiles of the monometallic catalysts, Cu/H and Mo_x_C/H, for which the desorption of CO_2_ is observed at 340 °C and at temperatures above 400 °C, respectively, seem to indicate that the CO_2_ desorption peaks of the bimetallic samples are linked to Cu-Mo sites. Moreover, the lower temperature at which these peaks appear in the bimetallic samples compared to monometallic catalysts can be related to the higher selectivity to methanol of bimetallic catalysts.

Cu^+^-Mo_x_C sites have been previously ascribed to increased selectivity to methanol [31], and on the CO_2_-TPD, they should correspond to higher desorption peaks compared to Cu-Mo_2_C. However, the catalysts also contain MoO_x_C_y_ and possibly hydrides; hence, it is not possible to assign unambiguously the desorption peak. Nevertheless, the absence of the latter peak on rGO and CNT and the higher temperature at which the first desorption peak appears suggest the presence of different adsorption sites on the catalysts depending on the supports.

Finally, selected catalysts were characterised by XRD after reaction and shown in Figure 1B. The XRD pattern of the spent CuMo_x_C/H and CuMo_x_C/rGO catalysts are different to those of the fresh catalysts, and they now display sharper XRD peaks at 2θ of 34.3°, 37.7°, and 39.4° attributed to the β-Mo_2_C phase, although contribution of the MoO_3_ at 2θ of 34.3° cannot be disregarded. The spent catalysts synthetized under inert atmosphere show intense and sharp peaks at 2θ of 35.4°, 36.3°, 36.8°, 42.2 °, 53.0°, and 53.5° for CuMo_x_C/H-He, which correspond to the diffraction peaks of the MoO_2_. The pattern of the spent CuMo_x_C/H-700He catalyst also displayed the typical diffraction pattern of the oxide, but still some contributions ascribed to the β-Mo_2_C phase can be observed.

In contrast, the XPS analyses of the spent CuMo_x_C/H shown in Table 1 only showed one contribution of MoO_3_, and the calculated Mo/C ratios are lower than for the fresh samples. This, along with the reaction results that showed a stable conversion and selectivity, seems to indicate that the β-Mo_2_C is preserved and that larger particles are formed. Nonetheless the unavoidable passivation layer can be responsible for the XPS results that displayed MoO_3_. The methanol yield obtained in the present work with CuMo_x_C/H, shown in Table 2, is comparable to the values reported for Cu supported on other carbon supports such as carbon nanofiber/ZrO_2_, at 180 °C and 30 bar and also for co-doped nanospheres at 220 °C and 20 bar, for which 0.2 μmol g^−1^ s^−1^ and 0.24 μmol g^−1^ s^−1^, respectively, were observed [32]. Furthermore, the results obtained are higher than those reported for other systems of the type Cu/ZnO or Cu/ZrO_2_, which offered 0.08–0.09 μmol g^−1^ s^−1^ yield to methanol at 250 °C and 20 bar [33].^.^ It is worth mentioning that in the present case, the reaction temperature was lower, e.g., 150 °C, and therefore, the activity could be even higher at higher temperature.

## 4. Conclusions

The synthesis of copper-molybdenum carbide over a high surface area graphite has been studied using different temperatures and atmospheres. In addition, the synthesis at 600 °C and H_2_ has been also performed over reduced graphene oxide and carbon nanotubes as supports, for the sake of comparison. The results indicated that the supports employed can act as sources of carbon for the formation of carbides. According to the results, the best synthetic temperature under H_2_ for the graphite support was 600 °C, which allowed to obtain a higher proportion of molybdenum carbide and oxycarbide, while the treatment under He is not suitable for carburization using the tested supports.

Moreover, the results showed that a carbon support such as the commercial high surface area graphite, HSAG400, is better to obtain molybdenum carbide probably because of its higher ratio of reactive carbon compared to lab-prepared graphene oxide, as observed in Raman. On the other hand, the smaller these particles are, the easier it is to carburize them, which is reasonable considering that the support is the source of carbon. Moreover, the CO_2_-TPD results seemed to confirm the Cu-Mo interaction.

It is worth highlighting that the catalyst prepared at 500 °C provided similar conversion as that prepared at 600 °C, since one of the drawbacks of carbides is the high activation temperature which increases the operational costs of the catalysts.

## Figures and Tables

**Table 1:** XPS Binding energies (eV), concentration of species in brackets and atomic ratios.

Catalyst	MoO_3_	Mo_2_O_5_	MoO_2_	MoO_x_C_y_	Mo_x_C	Mo^0^	Mo/C	Cu/Mo
	Mo^+6^	Mo^+5^	Mo^+4^	Mo^+δ^	Mo^+2^			
CuMoxC/H550	232.4 (20)	-	-	229.6 (34)	228.6 (44)	-	0.014	0.325
CuMo_x_C/H	-	231.1 (10)	-	229.4 (39)	228.6 (51)	-	0.013	0.231
CuMo_x_C/H-700	232.1 (19)		-	229.6 (13)	228.5 (22)	227.9 (44)	0.036	0.327
CuMo_x_C/H-He	232.4 (55)	231.2 (33)	-	229.6 (12)	-	-	0.013	0.484
CuMo_x_C/H-700He	232.6 (68)	231.2 (6)	-	229.4 (16)	228.5 (10)		0.010	0.216
CuMo_x_C/rGO	232.1 (19)	-	230.4 (11)	228.9 (32)	228.2 (37)	-	0.012	0.228
CuMo_x_C/CNT	232.3 (17)	231.0 (15)	-	229.3 (39)	228.4 (28)	-	0.012	0.224
CuMo_x_C/H-PR	232.4 (100)	-	-	-	-	-	0.008	0.545

**Table 2:** Methanol yield obtained with the tested catalysts.

Catalyst	CH_3_OH Yield (μmol/s⋅g)
Cu/Mo_x_C/H-500	0.182
Cu/Mo_x_C/H-550	0.181
Cu/Mo_x_C/H	0.205
Cu/Mo_x_C/H-700	0.150
Cu/Mo_x_C/H-He	0.053
Cu/Mo_x_C/H-700He	0.077
Cu/Mo_x_C/CNT	0.140
Cu/Mo_x_C/GOr	0.169

## Supplementary Materials

The following supporting information can be downloaded at: https://www.mdpi.com/article/10.3390/nano12071048/s1, Figure S1: Raman spectra of the supports. Figure S2: CO_2_-TPD SUPPORTS.
