# Supplementary figures and images for: Effect of the Carbon Support and Conditions on the Carbothermal Synthesis of Cu-Molybdenum Carbide and Its Application on CO2 Hydrogenation to Methanol
Source: Nanomaterials (Basel). 2022 Mar 23;12(7):1048. doi: 10.3390/nano12071048 (PMC9000400; doi:10.3390/nano12071048)

## Supplementary Material

**Figure S1.** Raman spectra of the supports.

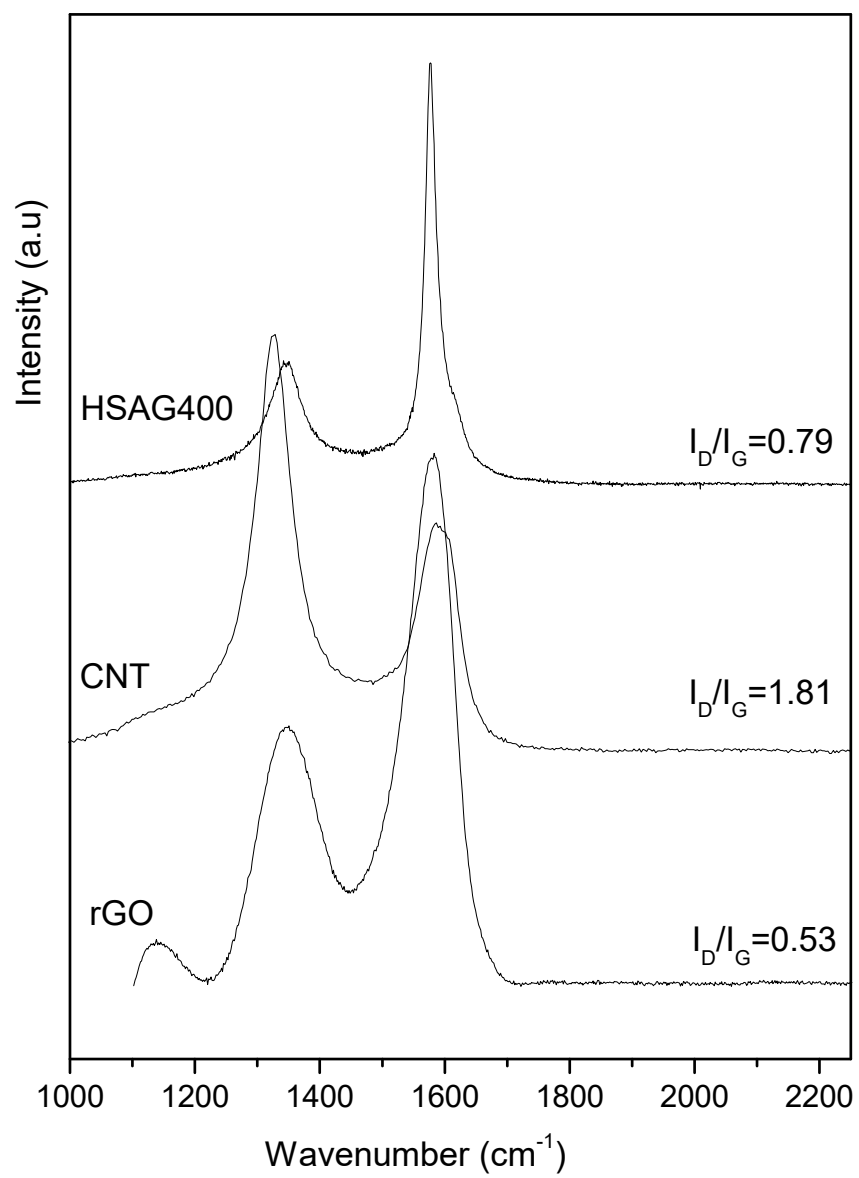

**Figure S2.** CO<sub>2</sub>-TPD supports.

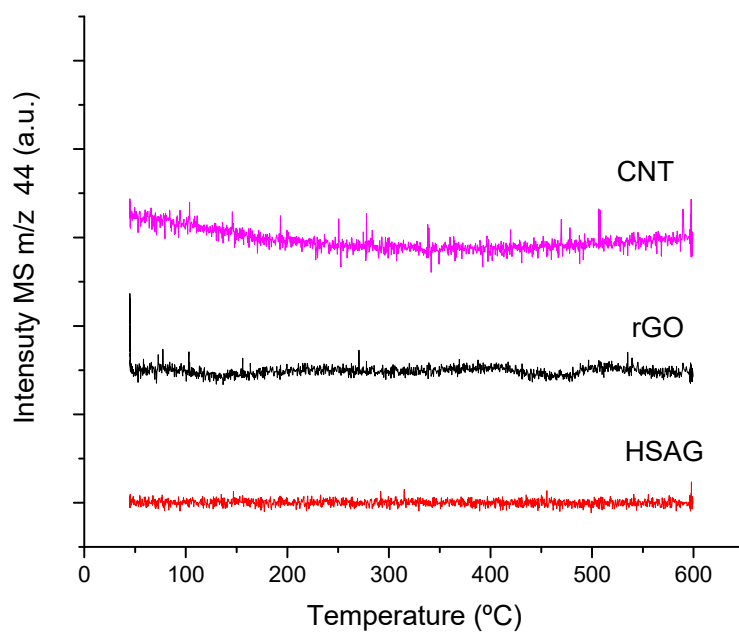

Supplement: Supplementary file 1 [file nanomaterials-12-01048-s001.zip › nanomaterials-1638181-supplementary.pdf]
